# High-quality *Acinetobacter* genomes recovered from combat wounds via metagenomic sequencing resemble cultured isolate genomes

**DOI:** 10.1128/spectrum.01876-25

**Published:** 2025-11-25

**Authors:** Jeffrey A. Kimbrel, James B. Thissen, Felipe A. Lisboa, Shalini Mabery, Crystal J. Jaing, Eric A. Elster, Seth A. Schobel, Nicholas A. Be

**Affiliations:** 1 Physical and Life Sciences Directorate, Lawrence Livermore National Laboratory 4578, Livermore, California, USA; 2 Surgical Critical Care Initiative (SC2i), Uniformed Services University of the Health Sciences (USUHS) 1685, Bethesda, Maryland, USA; 3 The Henry M. Jackson Foundation for the Advancement of Military Medicine, Inc. 44069 , Bethesda, Maryland, USA; 4 Walter Reed National Military Medical Center 8395, Bethesda, Maryland, USA; University of Manitoba, Winnipeg, Canada

**Keywords:** metagenome assembled genome, *Acinetobacter baumannii*, metagenomics, wound

## Abstract

The ability to accurately characterize wound pathogens is critical to informing clinical decisions for wound infections with complex treatment requirements. *Acinetobacter baumannii* is an impactful nosocomial pathogen in combat wounds and civilian hospital-acquired infections. An informed understanding of the phylogenetics and epidemiology of *A. baumannii* infections in military and civilian environments could guide approaches that improve antibiotic treatment regimens for both military and civilian patients. Whole-genome data for bacterial strains can be difficult to obtain due to challenges in culturing isolates from preserved military specimens. Metagenomic sequencing and assembly create opportunities for genomic analysis of pathogens directly from clinical specimens. The ability to perform comparative analyses between metagenome-derived genomes and culture-derived genomes would support a range of comparative bacterial genomic studies. Wound tissue biopsy and effluent samples from combat injuries were subjected to metagenomic sequencing and assembly. In total, 42 microbial metagenome-assembled genomes (MAGs) were obtained directly from metagenomic sequence data, 36 of which were designated “high” quality. Thirty of these genomes corresponded to *Acinetobacter*, with 29 mapping specifically to *A. baumannii*. Other observed genera included *Bordetella*, *Citrobacter*, *Escherichia*, and *Pseudomonas*. Single-copy and multi-copy orthologs were identified across *Acinetobacter* MAGs and publicly available isolate genomes derived from military and civilian sources. Both MAG and military isolate genomes were annotated with antimicrobial resistance data, and MAG genomes were statistically comparable to genomes obtained from isolates. Our results highlight the potential of *de novo* metagenome assembly for enabling high-resolution characterization directly from clinical specimens, thereby improving diagnostic precision, guiding antimicrobial stewardship, and enhancing understanding of pathogen evolution across diverse healthcare and battlefield environments.

**IMPORTANCE:**

The ability to comprehensively and accurately characterize microbial pathogens in wound infections is critical to efficacious treatment and is especially important in the context of complex battlefield injuries. Our study shows that high-quality metagenome-assembled genomes can be obtained from shotgun metagenomic sequence data for military-relevant wound pathogens including *Acinetobacter baumannii*. We demonstrate that these metagenome assemblies are directly comparable to genomes derived from cultured isolates, thereby supporting the ability to generate genome-level data sets from non-culturable biospecimens and perform comparative assessments that inform future approaches for improving diagnostic precision in military and civilian wound care.

## INTRODUCTION

Wounds resultant from combat injuries are subject to a wide range of unique clinical complications. Microbial contamination has always represented a significant source of morbidity and mortality in combat wounds (1). This remains true in wounds suffered after the post-antibiotic modern era of military conflicts (2, 3). Over 53,000 military service members were wounded in action from 2001 to 2020 across Operation Iraqi Freedom (OIF), Operation New Dawn, Operation Enduring Freedom (OEF), Operation Inherent Resolve, and Operation Freedom’s Sentinel (4). The Department of Defense Trauma Registry (DoDTR) Trauma Infectious Disease Outcomes Study (TIDOS), a comprehensive initiative that assesses infection data from injuries in Iraq and Afghanistan, observed an incidence of infectious complications in 34% of 1,807 combat casualties from 2009 to 2012 (5). Similarly, a study of combat extremity wounds by the Surgical Critical Care Initiative demonstrated that 30% of wounds studied demonstrated >1 × 105 CFU/g tissue via quantitative bacteriology (6). Studies suggest that standard microbiological techniques underestimate true microbial abundance (7). Thus, it is likely that the true bioburden is even higher.

Observations following OIF indicated an increase in *Acinetobacter*-associated infection specific to military patient populations (8). Assessment of trauma-related infections on a U.S. Navy hospital ship found that *Acinetobacter* was responsible for 36% of the observed infections (9). Multidrug-resistant (MDR) infections in combat injuries are particularly confounding, with negative impacts on patient progression and outcome, as management of care is rendered significantly more complex. A TIDOS study of trauma patients observed MDR infection events in 25% of cases, with *Acinetobacter* infection representing 39% of these instances (10). Our previous studies confirm the elevated incidence of *Acinetobacter* in combat extremity wounds and suggest that molecular detection of *Acinetobacter* is associated with poor wound outcomes (11), indicating that more detailed molecular and genotypic assessment of Acinetobacter is warranted.

Wound infection, in general, and *Acinetobacter* infection specifically influence wound healing progression and outcomes. It is likely that the incidence and progression of these infections differ between military and civilian environments due to fundamental distinctions in the nature, severity, and geolocation of injury. If genotypic differences in infecting bacterial strains of *Acinetobacter* species exist between military and civilian contexts, this could inform methods for managing such infection under defined scenarios. Even with competent care from experienced surgeons, some wounds fail to heal (12–14). The addition of more complete prognostic or diagnostic information on infection and antimicrobial resistance could support decision-making regarding antimicrobial management and stewardship by infectious disease specialists in Role 4 Military Treatment Facilities (equivalent to civilian tertiary hospitals). These specialists are available as part of multidisciplinary teams caring for hospitalized surgical inpatients. Their expertise ensures effective infection control, antimicrobial management, and long-term follow-up, significantly impacting outcomes and recovery. Their close collaboration with surgeons helps balance surgical decision-making with infectious risk management, supporting optimal care for complex, combat-related wounds. Thus, the availability of more complete patient data regarding wound infection could be readily functionalized for benefit via appropriate clinical experts.

In-depth characterization of genotypes for infecting microbial species typically involves microbiological culture and isolation. Obtaining biospecimens and corresponding isolates from wounds derived from combat injuries is challenging due to protracted transport times and austere collection environments (15, 16), polymicrobial bioburden (17), the presence of contaminating biomaterial from the environment and military gear (18), and the administration of broad-spectrum antibiotics (19). Repositories for such biospecimens can support analyses, including for military relevant specimens; however, preservation of such materials is often not consistent with conditions required for culture of viable microorganisms.

An approach for recovering and annotating wound-relevant, complete microbial genomes that does not require culture and can be applied directly to wound samples, such as tissue or effluent, would facilitate the collection of relevant data from specimens in which culture is no longer practical or feasible due to the loss of microorganisms' viability in the sample matrix of interest. Direct assembly of microbial genomes from shotgun metagenomic sequence data is one such approach, in which whole genomic DNA is extracted from clinical samples, followed by sequencing, metagenomic assembly, and classification. This would also allow for retrospective analysis of archived sample material from which viable microorganisms can no longer be obtained. Such a knowledge base would enable identification of genomic signatures relevant to clinical outcomes, which could be ported to existing rapid molecular diagnostic techniques.

To support efforts implementing comparative genomics to inform the epidemiology of military wound infection, we undertook a study to test the hypothesis that metagenome assemblies derived from complex combat wound biospecimens can effectively be integrated with isolate-derived genomes. We further endeavored to assess whether such metagenomes could be functionally annotated, and whether the resultant annotations could be employed for functional comparative assessment using existing isolate genome data. Evaluation of these questions is critical to informing future data generation from repository or other non-culturable sample matrices. Thus, we aimed to test this workflow in military-derived samples. Toward this end, we applied metagenomic assembly of microbial genomes (20) to samples from a cohort of combat-wounded patients. Resultant data were examined in the context of publicly available whole-genome data and antimicrobial resistance profiles in bacterial isolates from both military and civilian-derived infections.

## RESULTS

### Sequence read QC and assemblies

Whole metagenome sequence data from tissue biopsy and wound effluent samples were obtained from combat-injured patients (Table S1). A total of 78 total wound samples were previously collected during wound debridement procedures (14), and the read-based analysis of these samples was previously described (17). Only wound samples with sufficient bacterial read counts and depth to assemble at least 90% of an *Acinetobacter* genome were selected for further evaluation. This was determined by calculating the minimum reads of length 2 × 300 required to achieve at least 8× coverage for an average 3.75 MB *Acinetobacter* genome. Samples with more than 45,000 bacterial reads were chosen, retaining half (39 out of 78) of the data sets. A whole metagenome assembly and binning workflow was applied individually to each of the 39 samples (Table 1). Most (35/39) of the final assemblies were greater than 2 megabases (MB), and the assemblies smaller than 2 MB had the lowest read count and had less than 85,000 read pairs.

**TABLE 1:** Assembly metrics showing the mean, standard deviation (SD), and range for the 39 assemblies

Metric	Mean	SD	Range
Assembly size	4,961,652	2,765,046	3,105–12,283,964
Bacterial read pairs	1,168,282	1,851,890	45,252–10,503,282
Scaffold count	670	769	2–2,856

### Contig binning, MAG generation/QC/taxonomy

**Fig 1:**
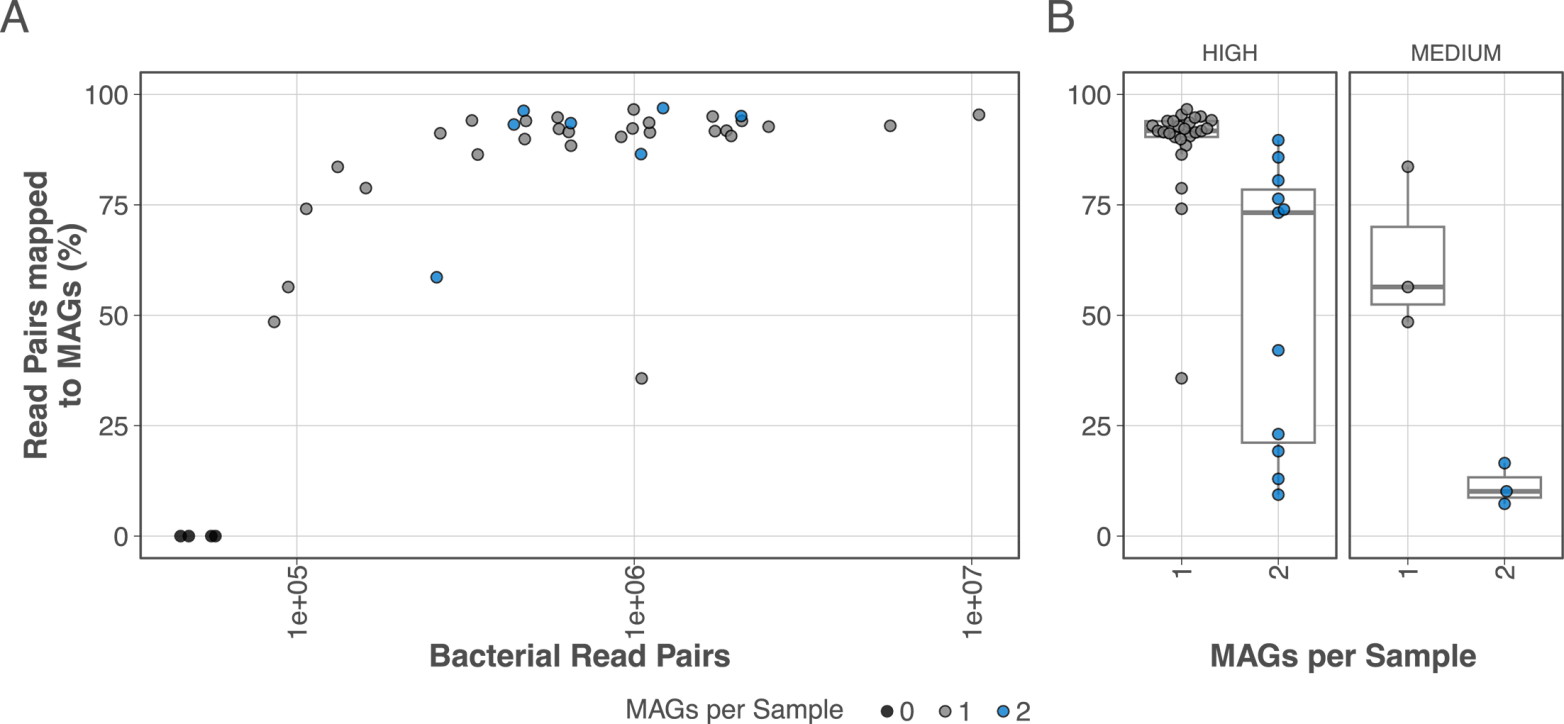
MAG metrics and read-mapping proportions show most read pairs mapped to MAGs in most samples. (**A**) All 35 samples with >85,000 read pairs successfully produced at least one medium- or high-quality MAG, while those with a low read pair count produced no MAGs. (**B**) MAG-based mapping statistics show that in most samples with only a single map, most of the reads in the sample mapped to that MAG*.*

The 39 metagenome assemblies underwent a binning, refinement, and reassembly workflow, successfully producing genome bins in all samples with >85,000 bacterial reads (35 samples), and failing to produce bins in the four assemblies with <85,000 reads. In total, 42 genome bins were produced and retained as metagenome-assembled genomes (MAGs; Fig. 1A). Twelve MAGs (Fig. 1B) were characterized as high quality (>90% complete, <5% contaminated with full-length 5S, 16S and 23S rRNA sequences); 24 were high quality but with at least one partial rRNA gene sequence; and six were classified as medium quality (>50% complete, <10% contaminated), according to the Minimum Information about a Metagenome-Assembled Genome (MIMAG) quality standards (21). Thirty-four of the 42 genome bins had tRNAs corresponding to 18 or more unique amino acids.

In 33 of the 35 samples with MAGs, more than 50% of the read pairs mapped to the MAGs, and 24 of the samples had greater than 90% read mapping (Fig. 1A). These results suggest a low species diversity at these sites, with most samples dominated by only one or two species. However, a high mapping percentage is not required for successful genome reconstruction from these samples, as high- and medium-quality MAGs were obtained from samples with as little as 9.4% and 7.3% read abundance, respectively (Fig. 1B).

### Taxonomy

Taxonomic evaluation was performed on each MAG using the Genome Taxonomy Database (GTDB; Table 2). Thirty of the 42 MAGs were assigned to *Acinetobacter* (29 to *A. baumannii* and one to *A. pittii*). In addition, nine MAGs were assigned to *Pseudomonas* (two *P. aeruginosa*, three *P. stutzeri*, three *P. monteilii*, and one *P. balearica*). Finally, one MAG each was assigned to *Bordetella*, *Citrobacter freundii*, and *Escherichia flexneri*.

**TABLE 2:** Summary of MAG GTDB taxonomies and MIMAG quality classifications.

Species	High	Medium
*Acinetobacter baumannii*	26	3
*Acinetobacter pittii*	1	0
*Bordetella* sp002261475	1	0
*Citrobacter freundii*	1	0
*Escherichia flexneri*	0	1
*Pseudomonas aeruginosa*	2	0
*Pseudomonas*_A *balearica*	0	1
*Pseudomonas*_A *stutzeri*	1	0
*Pseudomonas*_A *stutzeri*_AG	2	0
*Pseudomonas*_E *monteilii*_B	2	1

### Final downselection

For the subsequent comparative genomic analysis, the final list of MAGs was further restricted to *Acinetobacter baumannii* genomes with MIMAG “high” quality and at least 95% completion, resulting in a total of 23 MAGs.

### Orthogroup tree of final MAGs

**Fig 2:**
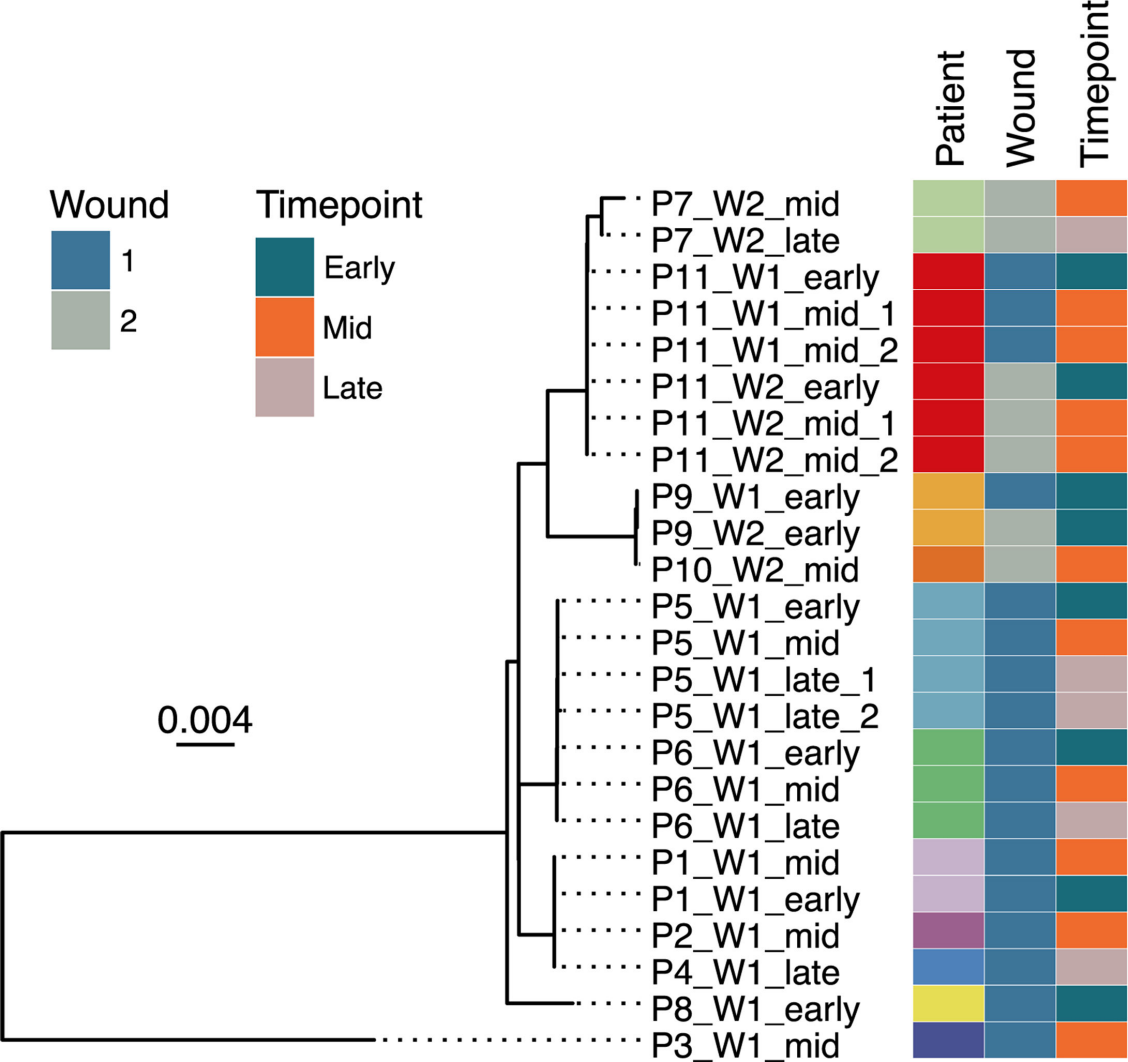
A phylogenetic tree of 105 ubiquitous, single-copy orthogroups for the 23 *Acinetobacter baumannii* MAGs. *A. pittii* MAG P3_W1_mid is included as an outgroup.

One hundred five single-copy orthologous gene groups (orthogroups) that were ubiquitous in all MAGs were used to create a phylogenetic tree (Fig. 2). There are several clusters showing high relatedness of MAGs, both between different MAGs from the same patient and wound, and between different patients. In all cases where MAGs were derived from specimens obtained from the same patient, these MAGs from the given patient are observed within the same cluster. There are instances (e.g., P9 and P11) where MAGs were obtained from different wounds within the same patient. Consistent with the observation above, these MAGs are observed within the same cluster. These results demonstrate the high degree of relatedness (and in some cases, likely identity) of microbial strains associated with the wounds from each given patient. Notably, this relatedness is also observed between distinct wounds within the same patient (P11), indicating multisite colonization by the same strain within a patient.

### Comparative genotypic assessment with military and civilian isolates

The 23 downselected *Acinetobacter* MAGs were compared to publicly available whole-genome sequences of cultured *Acinetobacter* isolates obtained from both military (origin of Iraq or Afghanistan) and civilian sources of hospital-acquired infections with the aim of resolving genotypes between MAGs and isolates of distinct origins (Table S2). Seventy-two and 326 isolate genome sequences were assessed from combat-associated (WRAIR multidrug resistance surveillance network [MRSN] and Walter Reed Army Medical Center [WRAMC]) and U.S.-based civilian injuries, respectively. To enable direct genome comparison of the isolate genomes to the MAGs, the 398 isolate genomes were subject to the same quality filtering and taxonomy criteria. Twenty-five isolate genomes had a CheckM completeness of less than 95% or contamination greater than 5%, and an additional 41 had a GTDB taxonomy other than *A. baumannii*. These genomes were removed, resulting in a total of 332 downselected isolate genomes (Table S3). Further, to eliminate the potential biases introduced by extensive hand-curation of the individual isolate genomes deposited in NCBI, the isolate genomes were reannotated for gene prediction and functional annotation with the same workflow applied to the MAGs (see Materials and Methods).

### Genome metrics

Comparisons of high-level genome assembly metrics showed that the MAGs were very similar to both the civilian and combat isolate genomes (Table 3). Three genome metrics showed significant group differences (ANOVA, BH-adjusted *P*-value < 0.05). A Tukey’s honest significant difference (HSD) test revealed that these differences were due to the civilian isolate genomes, with no significant differences observed between the MAGs and the combat isolates. The biological importance of the differences in these statistically significant metrics (e.g., 99.6% versus 99.9% completeness) is likely negligible. All genome types (MAG versus isolate) can be considered to be highly similar in their basic genome statistics, indicating that a comparative assessment between isolate- and combat-derived MAGs is statistically justifiable.

**TABLE 3:** Genome metrics (± standard deviation) and ANOVA comparison between MAGs and Isolates^*a*^

Metric	MAG	Civilian	Combat	ANOVA (BH)	η2	HSD
Completeness (%)	99.9 (0.2)	99.6 (0.6)	99.9 (0.2)	0.0000e + 00	8.563e-02	ab
Contamination (%)	0.3 (0.3)	0.7 (0.7)	0.3 (0.3)	1.9000e-04	5.485e-02	ab
Genome size (MB)	4.0 (0.2)	4.0 (0.1)	3.9 (0.1)	3.4531e-01	1.221e-02	–
L50 (MB)	0.1 (0.1)	0.2 (0.7)	0.1 (0.1)	1.0000e + 00	3.710e-03	–
Longest (MB)	0.3 (0.1)	0.4 (0.7)	0.4 (0.2)	1.0000e + 00	1.700e-04	–
Scaffolds	94.1 (60.9)	200.5 (191.2)	104.0 (50.7)	3.0000e-05	6.545e-02	ab

^*a*^ A post-hoc Tukey’s honest significant difference (HSD) test was run on significant BH-adjusted ANOVA results, and significant differences between combat-civilian and MAG-civilian are noted with an “a” or “b”*,* respectively; “–” indicates not significant. There were no significant HSD differences between MAG and combat. Effect sizes are reported as η2.

### Orthogroup conservation

To assess functionally relevant phylogenetic relationships between genomes extracted from wound specimens, genome-derived protein sequences from both MAGs and isolate genomes were obtained and clustered into a unified set of 7,975 orthogroups, each representing a complete set of orthologs identified through orthology inference. Analysis showed that 658 and 304 single-copy and multi-copy orthogroups, respectively, were identified as present in all 355 MAGs and isolate genomes, representing the intersecting subset of sequences with utility for inter-isolate comparative assessment. Single-copy orthogroups include orthologs found exactly once in all genomes, while multi-copy orthogroups were ubiquitous in all genomes but found in more than one copy in one or more genomes. Together, these 962 orthogroups represent the core genome for the *A. baumannii* MAG and isolate genomes.

**Fig 3:**
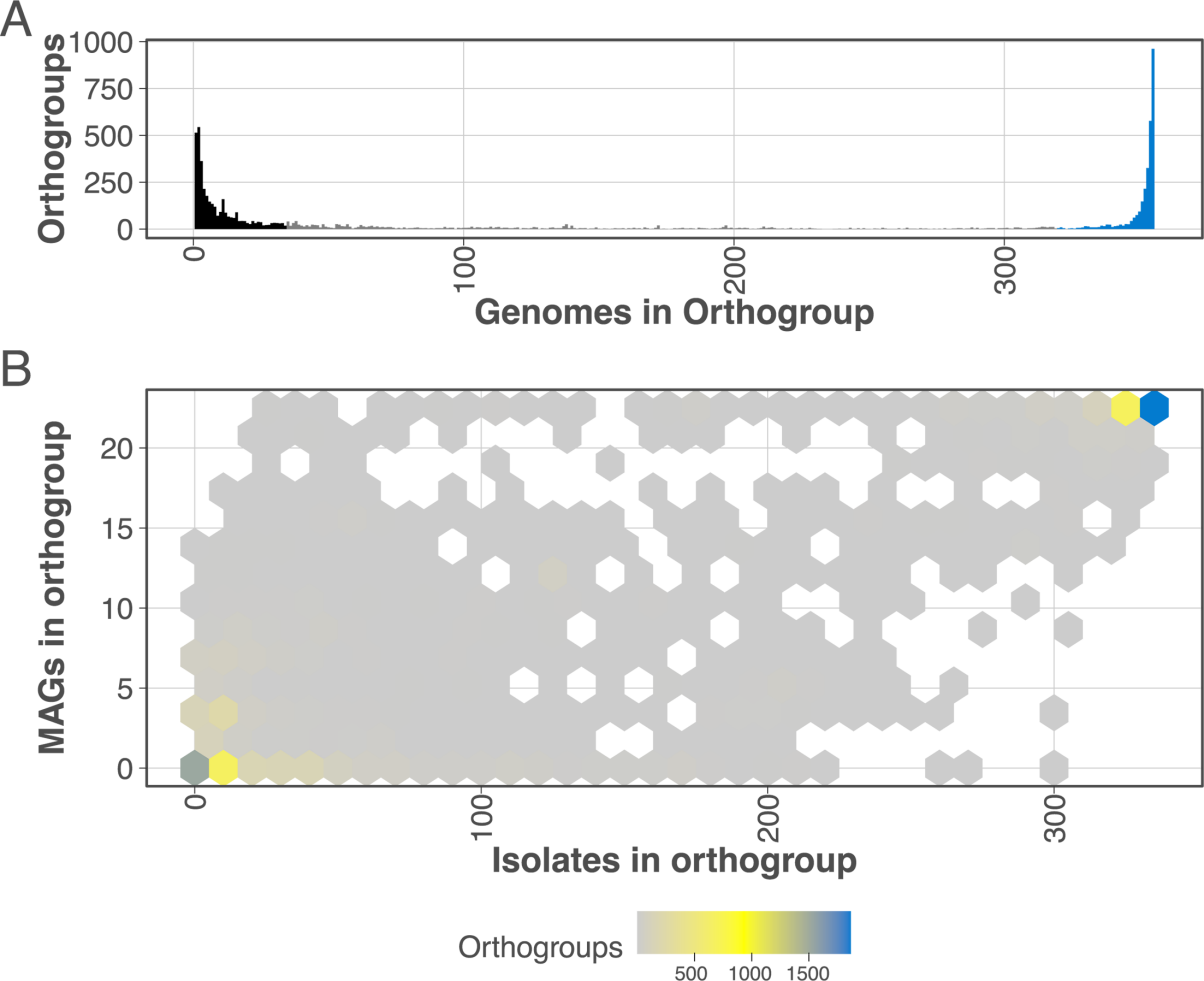
(**A**) Genome membership of each of the orthogroups shows that a majority are found in 90% or more (320) of the genomes (blue) or in less than 10% (35) of the genomes (black). (**B**) There are few orthogroups with membership consisting mostly of either MAG or isolate genomes, indicating there is not a systematic bias based on genome origin.

The cluster size of each orthogroup showed U-shaped bimodal distribution with a majority of the 7,975 orthogroups found in either more than 90% of the 355 genomes (2,828 orthogroups) or less than 10% of the genomes (3,504 orthogroups; Fig. 3A).

We next used the membership of the orthogroups to determine whether major differences in genomic content are attributable to the nature of obtaining a MAG versus an isolate genome sequence. These differences could be large-scale omissions of genes from the MAGs due to challenges in binning procedures, or the inclusion of contaminating sequences that are consistently placed into genome bins. To determine this, we plotted the MAG versus isolate membership count for all orthogroups (Fig. 3B). As Fig. 3A shows, a majority of orthogroups are present in either a few or most of the genomes. Similarly, Fig. 3B shows the abundance of orthogroups in the bottom left or top right of the heatmap, with few orthogroups present in the top left (indicative of a higher than expected skew towards a MAG membership) or the bottom right (indicative of an unexpected absence of MAGs from an orthogroup). Together, these results suggest that there is no systematic bias in genomic content between MAGs and isolate genomes, and there is no bias in gene content routinely either missed or added to the MAG genomes compared to the isolates.

### Functional annotation of *Acinetobacter* orthogroups in MAGs and isolate genomes

For further functional genomic comparison, all predicted protein sequences were functionally annotated for KEGG orthologs (KOs). In total, 2,216 unique KOs were identified across the 355 genomes. On average, 2,198 ± 48.02 KOs were annotated per genome, of which 1,749 ± 26.47 were unique within the genome (i.e., a KO assigned to only one gene). A total of 771 KOs were ubiquitous, found in all MAG and isolate genomes, and constitute the core functional genome of this group.

**Fig 4:**
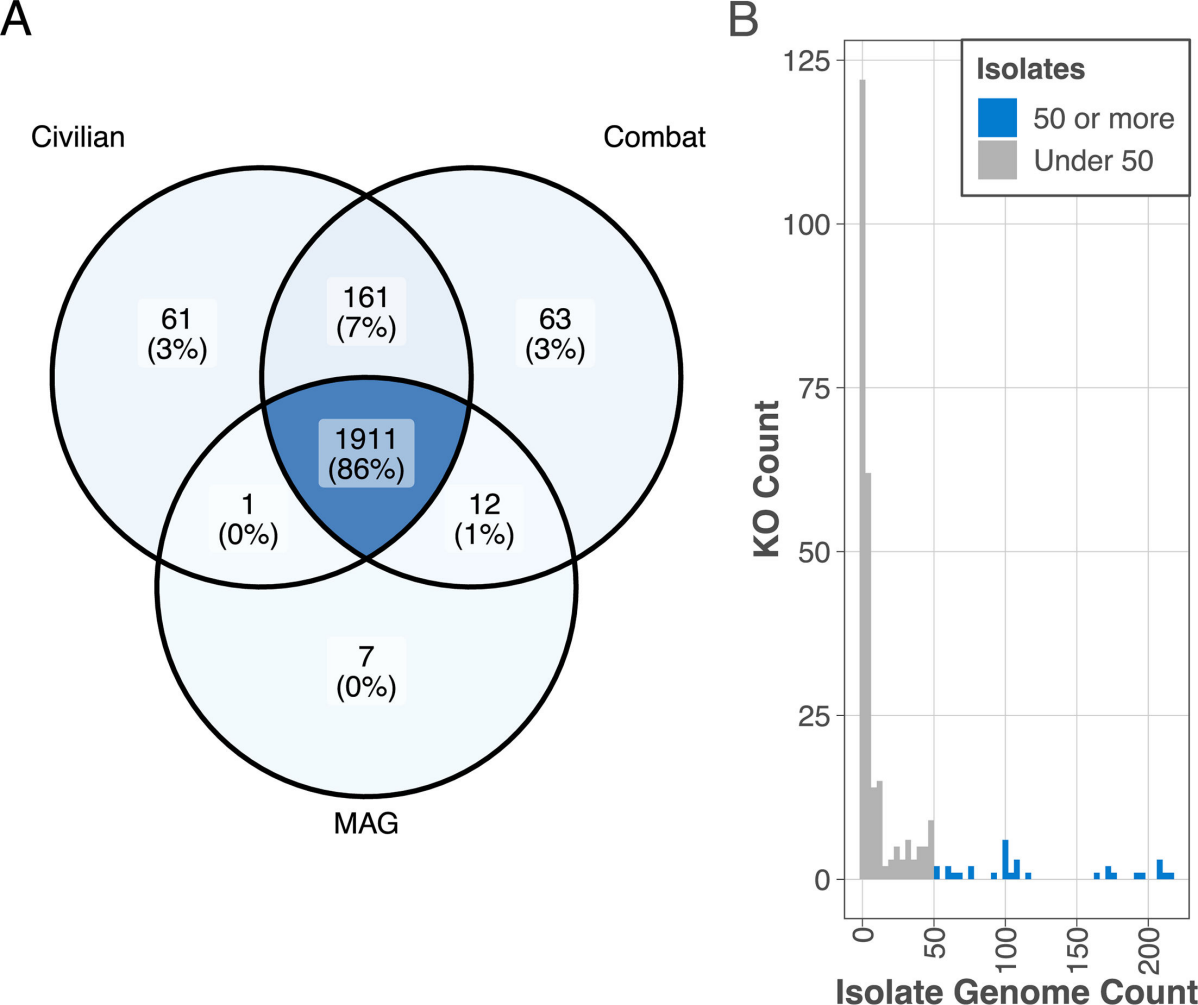
(**A**) Venn diagram showing the presence of KO terms in at least one genome of either the civilian or combat isolates, or a MAG. (**B**) Histogram of the counts of unique KOs (y-axis) missing from the MAGs, but found in at least one isolate genome (x-axis).

Although most (1,911 of 2,216) of the KOs were found in at least one genome from all three types (civilian, combat, or MAG; Fig. 4A), 285 KOs (12.9%) were found in either of the isolate types, but missing from all of the MAGs. This may be an artifact of the larger number of isolate genomes, as these non-MAG KOs are often found in only a few isolate genomes (Fig. 4B), suggesting there is no methodology-based reason for their absence in the MAGs.

There are, however, still 31 KOs missing from the MAGs but found in 50 or more isolate genomes (Fig. 4B; Table S4). When examining the orthogroups annotated with these KOs, none of the 31 orthogroups contained any MAG genes, indicating that these genes are absent from the MAG genomes rather than present but lacking KEGG annotations.

### Comparative genomic assessment of antimicrobial resistance and virulence factor genes

The Comprehensive Antimicrobial Resistance Database (CARD) (22) was used to annotate the presence of antimicrobial resistance (AMR) genes in the MAGs and isolate genomes. In total, 504 unique CARD Antibiotic Resistance Ontology (ARO) accessions were identified across the genomes. On average, there were 188.25 ± 5.95 AROs annotated per genome with no significant difference between the genome type. Seventy AROs were ubiquitous, found in all MAG and isolate genomes, and constitute the core AMR genome of this group. Additionally, 106 unique AROs were found in only one genome.

Overall, resistome annotation profiles derived from MAGs were similar to those observed from isolate reference genomes obtained from military sources, both with respect to detected genes and antibiotic resistance categories. Antibiotic resistance categories detected across multiple genomes of each genome type included resistance to carbapenem, cephalosporin, and streptomycin. Gene signatures for resistance included the multidrug efflux pumps *abeM* and *abeS* as well as numerous aminoglycoside-modifying enzymes. These results suggest that antimicrobial resistance signatures detected from metagenome-derived sequence could provide reliably comparable information to annotations derived from purified isolates.

The 355 downselected *A. baumannii* genomes were further annotated for virulence factor genes using the Virulence Factor Database (VFDB) (23). On average, each genome had 132.8 ± 10.05 loci annotated as virulence factors. There was no significant difference between the distinct genome types.

## DISCUSSION

These results demonstrate a comparative analysis of bacterial whole genomes obtained through *de novo* assembly of shotgun metagenomic data from combat wounds. These analyses enabled high-resolution genotypic analysis of wound pathogens without necessitating microbiological culture. Such an approach facilitates examination of non-culturable or fastidious microbes, in addition to facilitating retrospective microbial analysis of preserved samples no longer suitable for culture-based growth.

Comparative microbial genomic analyses are valuable for revealing distinctions between strains and subtypes that influence resultant severities of disease (24, 25), antimicrobial susceptibility (26, 27), and geospatial distribution and transmission dynamics (28, 29). Genome studies in nosocomial pathogens such as *Pseudomonas* have revealed genome-based differences in pathogenesis and environmental adaptation (30). Assignment of *Acinetobacter* to particularly virulent or resistant clades based on genome sequence could help assist in the prediction of future outbreaks through surveillance (31).

Recent studies focusing on *Acinetobacter* specifically describe the value of comparative genomics in evaluating antimicrobial resistance in environmental strains (32), performing epidemiological studies to evaluate geographic distinctions in lineage (33), and assessing mobile genetic elements with the potential to facilitate resistance acquisition (34).

Such analyses would be substantially supported by the capacity to perform comparative assessments between metagenomics and isolate-derived genomes. This has been demonstrated for a range of bacterial species, including *Acinetobacter*, to assess neglected diversity in non-traditional sampling environments (35, 36), antibiotic resistance genes in water sources (37), and resistance transmission in wastewater (38).

We extend such efforts to the realm of combat injury, where specimen analysis is complicated by a range of external factors, including austere environments, protracted patient transport times, and limited specimen preservation. Our results indicate that metagenome assembly and comparative methods can be applied to directly compare metagenomes derived from combat extremity wound biospecimens with whole-genome profiles obtained from publicly available cultured isolates.

Such analyses could support a range of comparative assessments, with utility for wound infection specifically and military medicine in general. Injuries suffered by service members in deployed combat environments are, in many respects, distinct from conventional traumatic injuries observed in the civilian world due to the energy intensity inflicting the injury, wound surface area, extent of contaminating environmental material, volumetric muscle loss, and substantial polytrauma (3, 39). Any corresponding microbial bioburden and associated infections may also be unique or disproportionately observed in these distinct populations and environments (40). Such distinctions could inform treatment, such as assignment of antimicrobial treatment regimens better suited for combat-associated colonization genotypes.

Our study demonstrates that whole metagenome-derived microbial genome assemblies may be used to identify bacterial strains, functional genomic characteristics, and antimicrobial resistance-associated genomic loci that may be distinct between categories of injuries. A primary limitation of this study is that paired cultured isolates were not available for the same preserved tissue specimens from which the metagenome assembles were derived. While it would be possible to perform a generalized study of this nature in a prospective civilian context, this study focused specifically on data resultant from the unique patterns of battlefield injury. Expanded studies will employ the genotypic features derived from methods described here to facilitate future predictive platforms that may inform clinical decisions for future battlefield injuries. Such studies will further benefit from rapid current and future advancements in metagenome analytics, including AI/machine learning-enabled assembly and annotation platforms (41). Results from this study, demonstrating that assembled metagenomes derived from complex, combat injury-derived sample matrices can be integrated with isolate-derived genomes for comparative analyses, will inform future assessments that leverage state-of-the-art, increasingly meta-aware methods to further improve accuracy and performance.

An additional limitation of the data sets implemented for this study includes the use of short-read sequence data. Advancements in long-read sequencing have, and will continue, to improve capacity for completing plasmids and annotating critical microbial genomic loci (42). The current study, however, demonstrates the promise of the described approach, which will be further amplified as technical methods improve in this field.

The ability to sequence, assemble, and annotate the genomes of wound-infecting microbial strains directly from clinical specimens would result in a substantial expansion in the capacity to generate annotated genomic data sets that track and help predict the evolution of military infectious disease. There are numerous examples of existing repositories for military service–derived tissues with infection relevance, such as those maintained by the Uniformed Services University Surgical Critical Care Initiative (SC2i) (43) and the Joint Pathology Center (JPC) (44), the nation’s oldest tissue repository. Specimens within such repositories are not necessarily suitable for microbial culture due to fixation and historical storage time/methods. This study demonstrates the feasibility of producing high-quality, low-contamination genomes directly from such specimens, where the resulting MAGs can be used in comparative genomic analyses alongside genomes derived from cultured isolates. Future expanded application of such methods would allow for comprehensive phylogenetic assessment of historical samples.

Studies in civilian medicine have demonstrated success in assessing historical trends of infectious disease (45, 46) and could provide comparable insight in military medicine. The current study demonstrates the feasibility of implementing comparative genomic approaches based on shotgun metagenomes derived from biospecimens from which microorganism culture is no longer viable. Expanded implementation of these methods will improve molecular epidemiological knowledge for assessment of wound infection patterns in similar repository specimens for past military conflicts. Additionally, such analyses will facilitate the prediction of evolving risks in future conflicts and combat theaters by expanding the quantity and breadth of microbial genomes accessible for comparative analyses. It is expected that combat extremity wounds will persist in future conflicts. An improved understanding of the evolution of military-relevant infections will facilitate selection of molecular diagnostic features, such as virulence or resistance signatures, that are most likely to be relevant in these challenging future environments.

## MATERIALS AND METHODS

### Sample collection and processing

Samples were previously collected from U.S. service member patients injured in Iraq and Afghanistan in compliance with all federal regulations governing the protection of human subjects and informed consent (Walter Reed National Military Medical Center Institutional Review Board protocol #352334). Sample handling procedures were carried out as previously described (3, 11, 14). Briefly, specimens were obtained during surgical debridements performed prior to wound closure. Tissue biopsy specimens were obtained from the wound center. Effluent specimens (fluid from the wound site) were sampled from the negative-pressure wound therapy canister. Specimens were stored at −80°C. Sample extraction, library preparation, and sequencing were performed as previously described (17). Briefly, genomic DNA was extracted from 10 to 30 mg tissue or 200 µL liquid effluent (cador pathogen kit, Qiagen) according to the manufacturer’s instructions. Extracted DNA was prepared for sequencing using the Nextera DNA Flex library preparation kit and sequenced via the NextSeq 500 (300-cycle high output kit V2, Illumina).

### Metagenomic read classification

Following shotgun metagenomic sequencing, the reads were filtered to include only those of suspected bacterial origin using the Livermore Metagenomics Analysis Toolkit (LMAT) pipeline to search for taxonomic identifiers associated with *k*-mers found in a corresponding reference genome database (47, 48). Only reads with *k*-mers identified in the “Bacteria” domain were retained for further analysis.

### Assembly and quality assessment

Bacterial reads were assembled using SPAdes v3.13.0 (49) with *k*-mer values of 21, 33, and 55. To obtain contig coverages, sequence reads were mapped to the contigs using bwa v0.7.17-r1188 (50). Resultant contig sequences were binned with MaxBin v2.2.6 (51), MetaBat v2.12.1 (52), and Concoct v1.0.0 (53). MaxBin parameters were min_contig_length = 1000, and the 107 gene marker set was used. MetaBat parameters were minContig = 1500, minCV = 1.0, minCVSum = 1.0, maxP = 95%, minS = 60, and maxEdges = 200. Default settings were used for Concoct, where LENGTH_THRESHOLD = 1,000 and KMER_LENGTH = 4. Genome bins obtained from these three algorithms were subsequently refined using the bin_refiner tool implemented in metaWRAP v1.3 (54), with a minimum CheckM v1.1.3 (55) completion of 50% and a maximum contamination of 10%. Sequence reads aligning to each bin were then recruited from the original read set and were subsequently reassembled with SPAdes v3.13.0. Original and reassembled genome bins were assessed for quality with QUAST v5.0.2 (56) and checkM v1.1.3 (55), and bins with a completeness score greater than 50% and contamination less than 10% were retained as metagenome assembled genomes (MAGs). MAG sequences were further searched for unique tRNA anticodons with tRNAscan-SE v2.0.12 (57), and for 16S, 23S, and 5S rRNA genes using Infernal v1.1.5 (58). These MAGs were scored according to MIMAG standards (21) as either medium or high quality based on their completion/contamination metrics, the count of tRNAs, and the presence of rRNAs.

### Cultured isolate genomes

The NCBI Pathogen Detection Browser for Isolates (https://www.ncbi.nlm.nih.gov/pathogens/isolates/, accessed 2020-02-10) was searched using the query string ‘taxgroup_name:“Acinetobacter baumannii”’ to obtain a list of *A. baumannii* isolates with genome sequences. These were further filtered to identify isolates of clinical and combat origin. NCBI accession identifiers are included in Table S2.

### All MAG and isolate genomes

Isolate and MAG genomes were assessed for quality with checkM v1.1.3 (55) and assigned taxonomy with the GTDB reference version r202 (59) using GTDB-Tk v1.5.0 (60). Genomes were retained if they had a CheckM completeness >95% and contamination <5% were assigned a GTDB taxonomy of *Acinetobacter baumannii*.

Predicted protein sequences were obtained for each genome by scanning with Prodigal v2.6.3 (61). The resulting predicted protein sequences were used for orthogroup analysis with Orthofinder v2.3.11 (62), functional annotation against the KEGG v102 database using KofamScan v1.3.0 (63) and the CARD v3.1.4 database with RGI v5.2.1 (22), and for BLAST searches against the VFDB “VFDB_setA_pro.fas” file downloaded on April 28th, 2022 (23). VFDB hits were further filtered to retain only those with an e-value less than 1e-7 and at least 50% sequence identity.

### Statistical analysis

Analysis of variance and Tukey’s HSD tests were done using the aov() and TukeyHSD() functions, respectively, from the stats package in R v4.2.3 (64). *P*-values obtained under multiple tests were corrected with a Benjamini-Hochberg adjustment and are denoted as “BH” when reported.

## Data Availability

The data underlying this study are not publicly available due to sensitivities regarding their generation from injured military service member cohorts. Data from the corresponding author are available on reasonable request and in accordance with applicable regulations and data usage agreements.

## SUPPLEMENTAL MATERIAL

Supplemental material spectrum.01876-25-s0001.docx Supplemental table legends.

Table S1 spectrum.01876-25-s0002.xlsx Average wound properties and locations.

Table S2 spectrum.01876-25-s0003.xlsx NCBI accession numbers and details.

Table S3 spectrum.01876-25-s0004.xlsx 332 downselected isolate genomes and 23 MAGs.

Table S4 spectrum.01876-25-s0005.xlsx 31 KOs missing from the MAGs but found in 50 or more isolate genomes.

ASM does not own the copyrights to Supplemental Material that may be linked to, or accessed through, an article. The authors have granted ASM a non-exclusive, world-wide license to publish the Supplemental Material files. Please contact the corresponding author directly for reuse.
