# Supplementary material for: High-quality Acinetobacter genomes recovered from combat wounds via metagenomic sequencing resemble cultured isolate genomes
Source: Microbiol Spectr. 2025 Nov 25;14(1):e01876-25. doi: 10.1128/spectrum.01876-25 (PMC12772318; doi:10.1128/spectrum.01876-25)
Supplement: Supplemental material — Supplemental table legends. [file spectrum.01876-25-s0001.docx]

# Supplemental Tables

Table S1 - Table with average wound properties and locations. supptbl-wound_metrics.xlsx

Table S2 - Table with NCBI accession numbers and details. *supptbl-Ab_genomes.xlsx*

Table S3 - Table with 332 downselected isolate genomes and 23 MAGs. supptbl-downselected_genomes.xlsx

Table S4 - Table with 31 KOs missing from the MAGs but found in 50 or more isolate genomes. supptbl-missing_KOs.xlsx
